# Accelerated Ray Launching Method for Efficient Field Coverage Studies in Wide Urban Areas

**DOI:** 10.3390/s23146412

**Published:** 2023-07-14

**Authors:** Josefa Gómez, Abdelhamid Tayebi, Carlos J. Hellín, Adrián Valledor, Marcos Barranquero, Juan J. Cuadrado-Gallego

**Affiliations:** Computer Sciences Department, University of Alcalá, 28801 Alcalá de Henares, Spain

**Keywords:** accelerated shooting and bouncing ray, propagation, ray launching, ray tracing

## Abstract

The implementation of a fast and efficient computer tool for field coverage studies in urban mobile radio systems is presented in this work. An accelerated and tailored ray launching method takes advantage of a ray tracing programmable framework optimized for massively parallel processing on GPUs. The PlotOptiX API is used to customize the code before applying the electromagnetic equations. The proposed code is described, and results are shown to demonstrate its correct operation. A high number of diffractions and reflections can be tracked in each ray from the transmitter to the receiver. In addition to the typical point-to-point simulation, measurement planes can also be set as receivers to provide fast predictions in wide urban areas.

## 1. Introduction

According to the literature, two main categories of ray-based deterministic models can be distinguished for radio wave propagation prediction: ray tracing based on the image theory concept, and ray launching based on the shooting and bouncing ray technique. Deterministic models, which are based on Maxwell’s equations, are preferred to empirical and semi-empirical models [1] because they provide more accurate predictions of signal propagation. Unfortunately, the computational requirements (memory and time) increase when the scenario under analysis becomes more complex. Consequently, many efforts have been made in the last decades to improve and accelerate these methods [2,3,4,5]. Moreover, in situations of non-line-of-sight, ray launching methods are chosen since, normally, ray tracing methods include only a limited number of specular reflections and diffractions [6].

The goal of this work is to implement an accelerated ray launching method based on a ray tracing programmable framework, capable of handling requirements specific to the radio wave propagation analysis. The proposed method can also provide fast predictions in wide urban areas by setting measurement planes instead of configuring the typical point-to-point simulation. It is worthwhile to point out that ray tracing is a popular and attractive technique in computer graphics due to its ability to produce a high degree of visual realism. The advantage of the proposed approach is the possibility of using a commercial ray tracing framework optimized for massively parallel processing on modern graphics processing devices called GPUs. Since the purpose of these frameworks is graphics visualization, the algorithms they provide must be tailored to calculate specular reflections on walls and diffractions on wedges of buildings. Concretely, the field calculation differs from a typical image generation performed with ray tracing algorithms. For example, the phase at the receiver and at every intermediate hit needs to be known for each traced path, as well as path segment lengths and their directions with respect to the object normals. Thus, a software stack needs to allow for easy access to the internal stages of the ray-tracing process. PlotOptiX [7] was used as a base for experiments. It is a Python package that enables combining components prototyped in a high-level language with the optimized workflow of the underlying OptiX [8] ray tracing engine. The OptiX engine handles compute-extensive tasks such as traversal of the scene and provides the GPU-oriented infrastructure for launching user-defined rays or executing custom code to determine the ray-object intersection and calculations when the intersection is found. PlotOptiX API allows for interaction with the OptiX engine from the level of the Python code. It handles initialization, provides functionality to define custom blocks of ray tracing code, and collects data required for further processing of the field coverage. In multi-GPU systems, PlotOptiX seamlessly handles the work distribution.

Once all the rays are obtained, Geometrical Theory of Diffraction/Uniform Theory of Diffraction (GTD/UTD) equations [9] are applied to obtain the received field at a certain point(s) or within a wide area efficiently and accurately (see Appendix A).

The NVIDIA OptiX ray tracing engine has been recently used in different tools for several applications such as particle-based simulations [10], threat detection systems [11], PET scans Monte Carlo simulations [12], X-ray transmission imaging [13], and electromagnetic characterization [14,15]. However, although previous works [14,15,16,17] have introduced the idea of using the OptiX framework to accelerate the ray tracing, the authors have not found any work in the literature that focuses on the calculation of the received power in broad areas using a measurement plane as a receiver. The most similar work is found in [14], where OptiX is used to perform the ray tracing and heatmaps of the received electromagnetic field can be obtained setting hundreds of receivers on the scenario. The novelty of the proposed work is that those heatmaps can be efficiently obtained by setting a measurement plane as a receiver instead of setting hundreds of dots as receivers on the scenario. In [15], an alike method is also described. However, it does not allow the computation of the received power on a measurement plane neither.

The main contribution of this paper is that in addition to the typical point-to-point simulation, the developed tool allows setting a measurement plane of any size to provide fast predictions in wide urban areas. Thus, extensive coverage simulations can be carried out almost instantaneously. Other simulation tools available such as NewFasant and Winprop [18] compute the received field in a grid of points, iteratively in several simulations, and then the obtained values are interpolated and shown on a measurement plane. In contrast, the idea behind this work is to identify all the possible rays that reach a certain measurement plane that has been previously established. The advantage of the proposed method is that only one simulation is required, reducing the total calculation time. To the best of our knowledge, the proposed code is the only simulator with this feature.

The rest of the paper is organized as follows. An overview of the developed code and the implementation details are presented in Section 2. The results are shown in Section 3. Finally, Section 4 presents future lines of work and conclusions.

## 2. Materials and Methods

### 2.1. Code Overview

**Figure 1:**
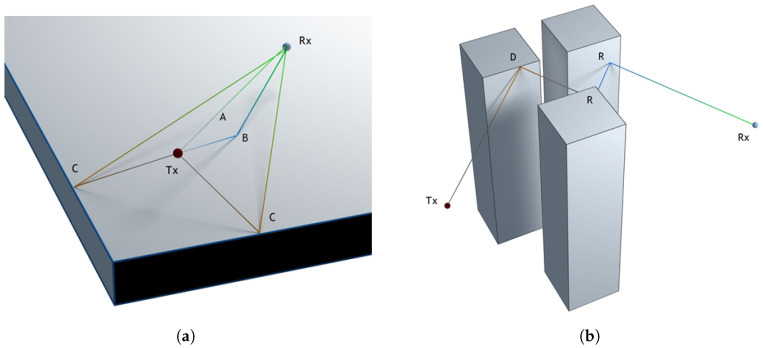
Simple scenes with visualizations of paths connecting the transmitter Tx and receiver Rx: (**a**) direct visibility (A), single reflection (B), and single diffractions (C); (**b**) path with diffraction on the wedge (D) and two reflections (R).

**Figure 2:**
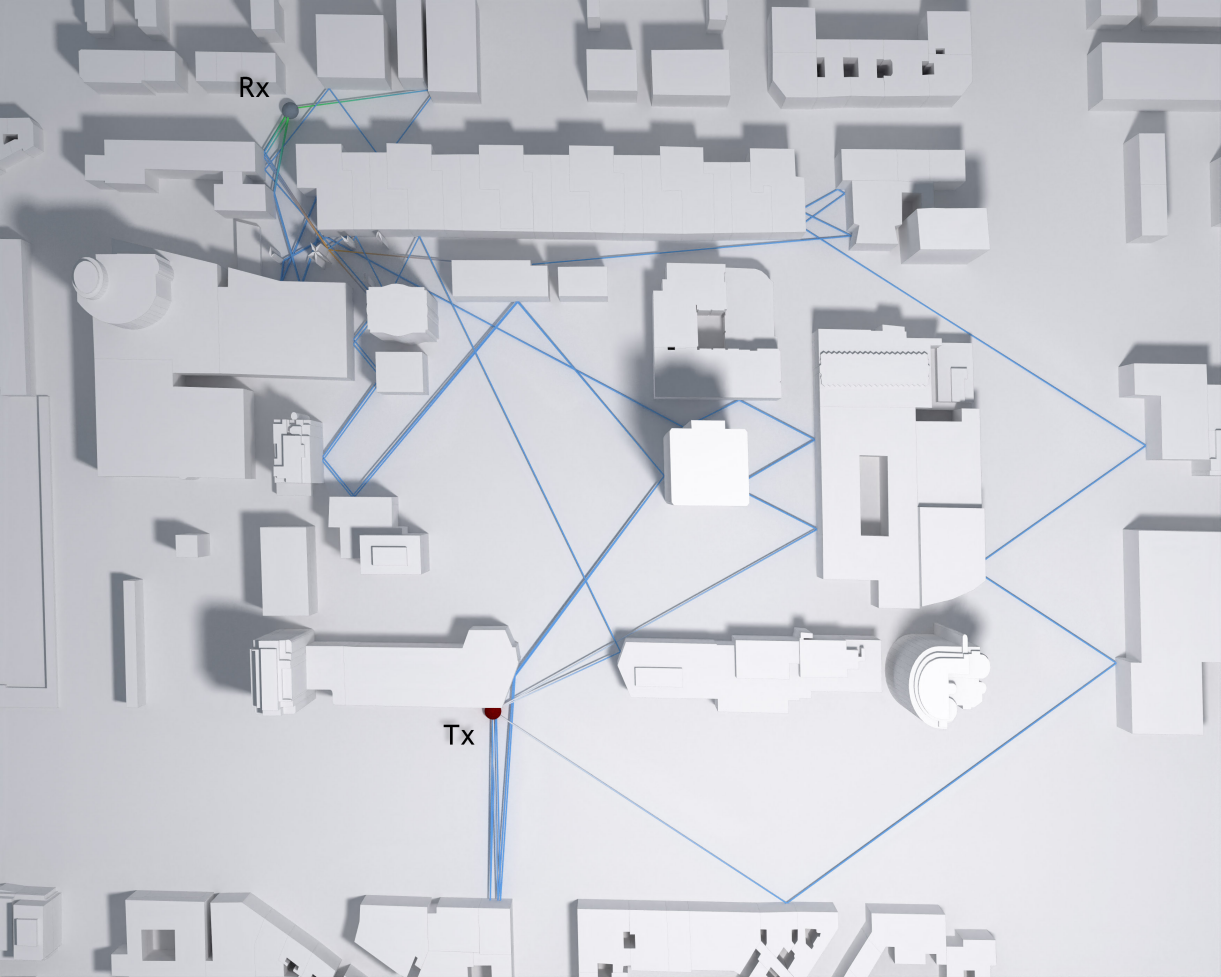
Non-line of sight situation in which a high number of reflections and diffractions are required to compute the received signal strength. The transmitter (Tx) is modeled by a red sphere, and the receiver (Rx) is modeled by a gray sphere.

Paths connecting the transmitter and receiver need to be found in the 3D scene for the calculation of the field at the receiver location. Paths can be in the form of single linear segments in case of direct visibility or can be composed of multiple segments due to possible reflections and diffractions on the scene objects on the way between the receiver and transmitter. As shown in Figure 1, reflections occur on flat faces, while diffraction happens on the wedges of objects. Figure 1a shows a simple example of the main types of rays: direct rays, reflected rays, and diffracted rays. A direct ray can be observed between the transmitter and receiver (path A). Path B represents a reflected ray, and path C represents two diffracted rays. As can be seen, both diffracted rays hit on wedges. On the other side, Figure 1b shows a ray transmitted from Tx, which is diffracted (D) and then reflected twice (R) before reaching the receiver Rx. Since there are no a priori assumptions on the course of successful paths, a large number of uniformly distributed rays starting from the transmitter need to be traced, while only a small fraction of them reach the receiver. Path segments are described with segment length, their 3D orientation, the identifier of each object and the primitive within the object (face or wedge) at the end of the segment, as well as the identifier of the event that occurred at the end of the segment (receiver hit, reflection, diffraction, or end of the path). This complete information is required for the later field calculation. Data are collected for each segment in each path starting from the transmitter, and only upon the completion of tracing all paths, the dataset is reduced to contain only successful paths arriving at the receiver. It is important to highlight that a large number of diffractions and reflections can be tracked in each ray from the transmitter to the receiver. A top view of Castellana Street, in Madrid, can be seen in Figure 2. It is worthwhile to remark that the simulations provide 3D results because the transmitter and receiver can be located at any height. Figure 2 shows an example of a non-line-of-sight situation in which four successful paths, each including fifteen reflections and diffractions, have been processed between the transmitter and the receiver in order to calculate the received signal strength.

**Figure 3:**
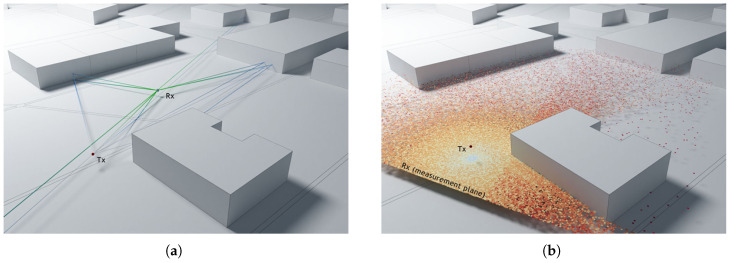
Implementation of the receiver (Rx): (**a**) sphere; each path connecting to the transmitter (Tx) is a unique sequence of wedge and face hits; (**b**) plane; all paths from the transmitter (Tx) are collected; each patch in the image represents a hit on the receiver plane (corresponding path not shown for clarity). The lightest color represents the highest received field strength, and the darkest color represents the lowest received field strength.

The transmitter is implemented as a point source of rays, while the receiver has to be a solid object or at least a plane to allow the ray tracing algorithm to find an intersection with it. Two options for the receiver are provided:

1. A sphere or various spheres that allow the calculation of the field at a narrow location(s), approximating the point-like receiver(s) with the small radius of the sphere(s) (see Figure 3a). Further reduction of saved paths is applied in this scenario to leave only one path for each unique sequence of object faces and wedges.
2. A plane that allows for the collection of large statistics of rays over the interesting area (see Figure 3b). This option is computationally more efficient due to the higher probability of hitting the receiver. The receiver is transparent and can be hit multiple times in a single path. The received power along the measurement plane can be displayed as shown in Figure 3b. This capability provides direct observation of coverage in a typical urban environment and takes into account the 3-D nature of buildings and other structures in the propagation environment.

Objects in the scene are composed of triangular meshes with cylinders along wedges, forming convex ridges that contribute to the diffraction. The cylinders on the wedges have relatively small cross-sections. Keeping the cylinders thin prevents a significant reduction of the face surfaces that could affect the reflection results, but it is necessary to give them volume so they can be detected in the ray tracing.

Finally, the field values at receiver locations are calculated from selected paths in the high-level Python code. In the case of multiple transmitter locations or frequencies, the above workflow is repeated for each configuration.

### 2.2. Implementation Details

**Figure 4:**
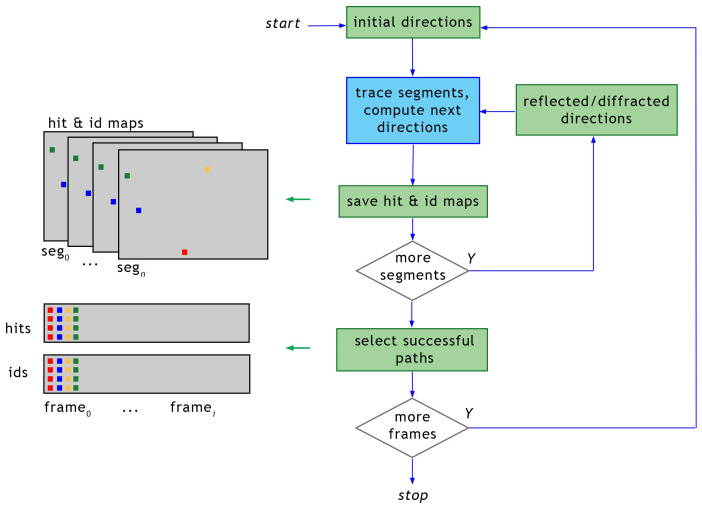
Scheme of multi-segment path tracing. Green blocks are the Python code responsible for collecting data from the GPU and filtering paths connecting the transmitter and the receiver. The blue block is the segment tracing code executed on the GPU (see Figure 5).

Tracing large amounts of paths may consume GPU memory beyond available resources. Thus, the workflow presented above is organized in frames. Each frame is a set of paths traced together. Paths can be composed of multiple linear segments, and tracing a frame of paths progresses in batches: one segment from each path per batch. Batches are launched repeatedly until all paths are terminated or reach the maximum number of segments (see Figure 4).

**Figure 5:**
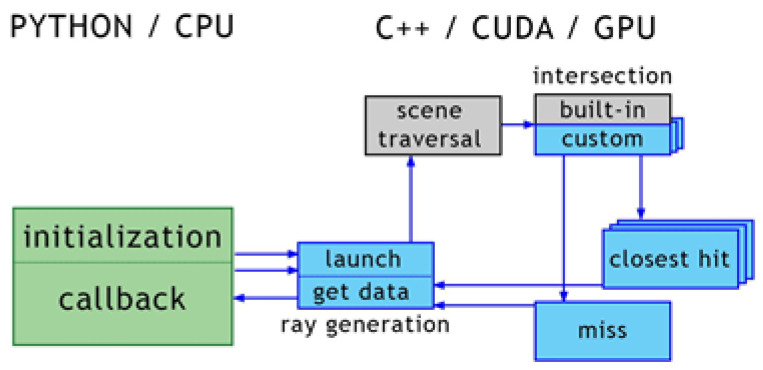
Scheme of a single segment tracing. The green block is the Python code executed on the CPU. This code interacts with the C++/CUDA kernels executed on the GPU using the OptiX framework via the PlotOptiX API. The gray blocks are OptiX built-in kernels, while the blue blocks are custom code kernels provided to the framework via PlotOptiX.

Then, data corresponding to paths arriving at the receiver are collected from buffers filled with results for all traced paths. Processing each segment in the batch follows the scheme shown in Figure 5. Parallel execution of tracing segments is not visible to the user code, and it is maintained by the OptiX framework. In addition, the distribution of work between available GPU devices is transparent to the user code and implemented in the PlotOptiX package. The framework provides building blocks for tracing path segments using custom user programs interconnected with built-in functions, as presented in Figure 5. Batches are interleaved with callbacks to the Python function responsible for the collection of processed segment data (described in the previous section), and initialization of segment directions for the next batch.

**Figure 6:**
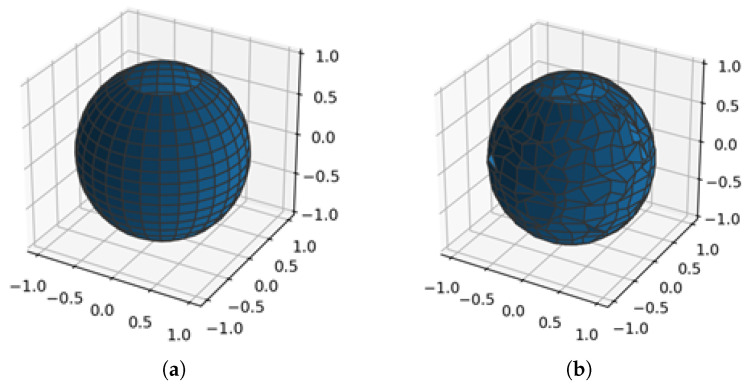
Distribution of the initial segment directions: (**a**) a regular grid (equal cell areas) is used in the first frame; (**b**) jitter is added to the regular grid in consecutive frames.

Initial segments start at the transmitter with directions passed to the ray generation program from the level of the Python code. Directions in the first frame are uniformly distributed over the grid, as shown in Figure 6. To improve performance, a limit on the maximum vertical angle can be applied, as the vertically starting paths may have negligible chances of reaching the receiver. Initial directions in the next frame have a random jitter of the size of the grid used in the first frame. Randomization allows for avoiding accidental synchronization of the regular direction distribution with a potential pattern of the scene meshes, which could bias the result.

Once the segment tracing is launched with the ray generation program, the scene is traversed to find the closest intersection of the segment with an object’s face or wedge. If no intersection is found, then the miss program is executed. The search uses bounding volume hierarchy (BVH) traversal and calculation of potential intersection positions with objects inside the volumes crossed by the segment. This process is implemented within the OptiX engine, which is shipped as a part of the official NVIDIA driver [19]. The exact implementation of BVH traversal and ray-object intersection location depends on the driver release as well as on the hardware on which the code is executed, e.g., ray-triangle intersections can be hardware operations on devices that support such a feature. Implementations of intersection formulas with user-defined geometry primitives (e.g., the receiver geometry can be a sphere in this work) are implemented as CUDA C++ kernels within the PlotOptiX package.

**Figure 7:**
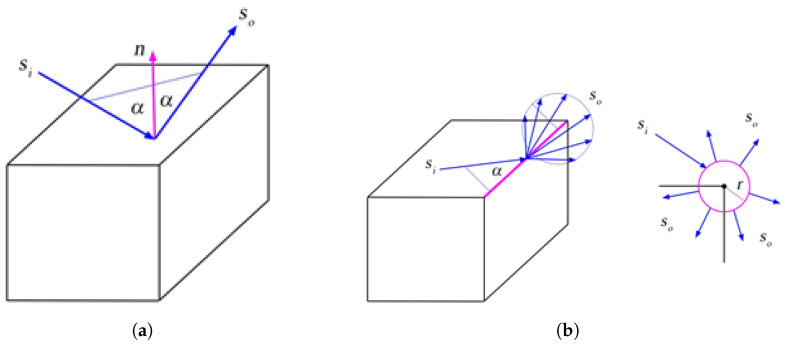
Reflection from the object’s face (**a**) and diffraction on the object’s wedge (**b**). Incoming segment $S_{i}$ and reflected outgoing segment $S_{o}$ are at the same angle with respect to the face normal *n*. The outgoing segment diffracted from the wedge is drawn from $S_{o}$, forming a right circular cone around the wedge with the apex angle α (Keller’s cone). In the ray tracing algorithm, a narrow cylinder with radius *r* is placed along the wedge to find the incoming segment’s intersection with the wedge. The outgoing segment is displaced to the cylinder’s surface to avoid repeated intersections with the wedge, as shown in the view along the wedge. Note also that it is possible to draw outgoing segments pointing inside the object.

In the next step, hit programs are executed for segments with an intersection found and miss programs for the remaining segments. These programs are, again, custom CUDA C++ kernels added to the PlotOptiX package, similar to the intersection kernels. Hit programs compute the direction of the consecutive segment and save it together with the identifier of the involved object and primitive. Values are stored in GPU buffers, at the address corresponding to the traced segment. Two different hit programs are implemented to handle direction calculations for hits on the object faces and wedges. The reflected direction is simply determined by the incoming segment direction, and the involved face is normal, while a diffracted direction is drawn as shown in Figure 7. There is a possibility to draw the diffracted direction that points inside the object. For code simplicity and performance reasons, such configuration is not tested immediately by the kernel code, and instead, the next traced segment is detected as pointing back to the face of the object, and the path is terminated. Another hit program supports the intersection with the receiver. It tags the segment with the receiver hit code and terminates the path (spherical receivers) or passes the unchanged segment direction as the next segment direction (transparent plane receivers). The miss program is executed if no intersection is found. The program tags the segment with the miss code, which also terminates the path.

## 3. Results

The proposed code has been tested and compared to other commercial tools. Concretely, some test cases have been run with NewFasant, a spin-off from the University of Alcalá that was acquired by Altair [20] in 2020. NewFasant has been validated in several research works [2,3,9,21]. The same test cases have been run with the developed code to measure its benefit in terms of computational time.

**Figure 8:**
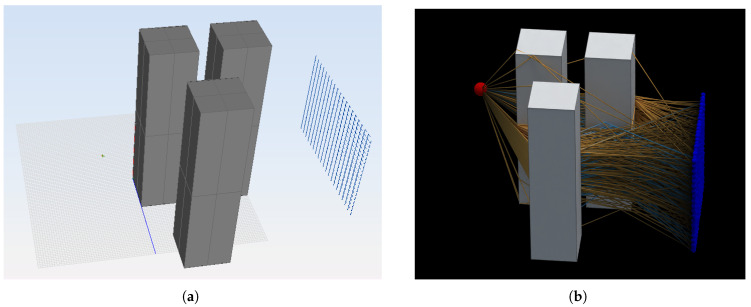
Scenario of the test case 1. The transmitting antenna is placed on the left, and the grid of receiving points is set on the right. (**a**) Screenshot from NewFasant. (**b**) Screenshot from the proposed tool.

The first analyzed scenario is shown in Figure 8, where a transmitting antenna has been set and a grid of 20 × 20 points has been placed to compute the received electric field in each one of the 400 points. The simulation frequency is 300 MHz, and both reflections and diffractions are allowed, with a maximum number of segments in each ray set to three. In both simulation tools, the transmitting antenna is modeled by means of a radiation pattern through a file that is read and processed by the code.

**Figure 9:**
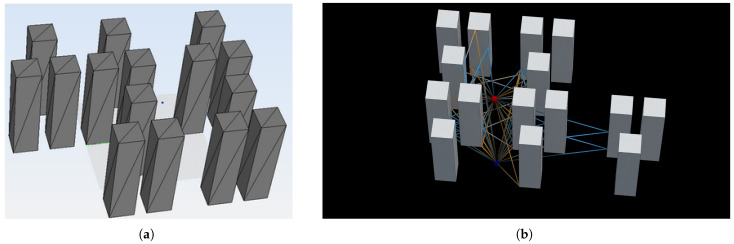
Scenario of the test case 2. (**a**) Screenshot from NewFasant. (**b**) Screenshot from the proposed tool. The transmitting antenna is represented with a red dot, and the receiving antenna is represented with a blue dot.

The second analyzed scenario is shown in Figure 9, where a transmitting antenna has been set, and only one receiver has been placed to compute the received electric field. The simulation frequency is 300 MHz, and both reflections and diffractions are allowed, with a maximum number of segments in each ray set to five.

It is important to point out that the size of the reception spheres for test cases 1 and 2 is calculated according to [22].

**Figure 10:**
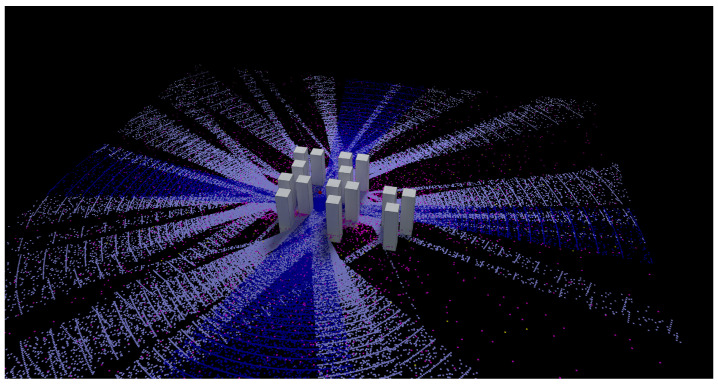
Results of the test case 3.

The third analyzed scenario is shown in Figure 10, where a wide area of 10,000 m^2^ has been simulated. In this case, the simulation frequency has been set to 1 GHz, and the transmitting antenna has been placed in the center of the scenario. A measurement plane has been set to capture all the rays launched from the source. It is important to highlight that, although the results are visualized on the plane, the rays are propagated in any direction (in 3D). A total of 1,874,779 paths have been identified and visualized in different colors: dark blue dots represent direct rays, light blue dots are reflected rays, pink dots are diffracted rays, and yellow dots represent rays that have been diffracted twice. It is important to point out that the electric field results can also be visualized, as shown in Figure 3b, where lighter colors indicate higher received field strength and darker colors indicate lower received field strength.

The computer used to obtain the results in Table 1 is an Intel Core i7 CPU, 3.8 GHz, and 32 GB RAM. The graphics card is Nvidia GeForce RTX 3070. According to Table 1, the required time to run the first test case using the proposed code is 36 s. However, NewFasant takes 2 min and 38 s to run the same test case. This huge difference is due to NewFasant applying the image theory (ray tracing) point by point in an iterative way, whereas the proposed code (ray launching) obtains all the rays at once from the transmitting antenna to the grid of points. Regarding the second test case, the required time using the proposed code is 3 s, and using NewFasant is 28 s. A noticeable reduction of time of 89% can be observed. Finally, while NewFasant is not able to run the third case, it can be simulated in 2 min and 53 s using the developed code.

**Figure 11:**
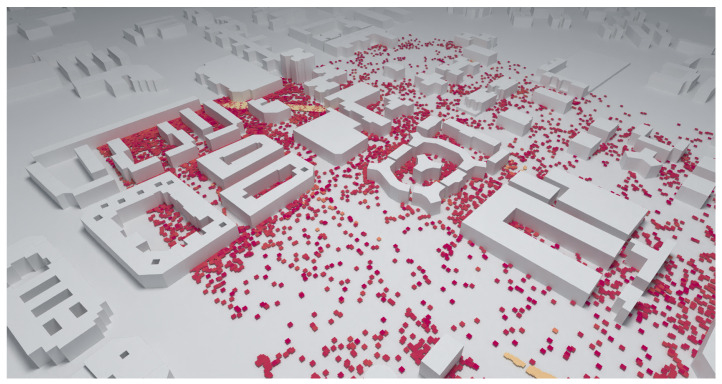
Results of a simulation carried out in a real scenario.

Figure 11 shows a real scenario in the city of Cartagena that was also analyzed in [14]. The authors indicate that 898 receivers were set, but they do not indicate anything about the runtime. The required time to perform the same simulation with the proposed tool using the measurement plane as a receiver is 23 s.

## 4. Conclusions

A fast-ray launching-based tool for field coverage studies has been presented. The proposed tool is particularly useful for analyzing wide urban areas. As shown in Figure 3b, Figure 10 and Figure 11 when the receiver is set as a measurement plane, coverage studies can be easily obtained based on the radio propagation losses or based on the received field strength efficiently. In addition, the proposed code takes advantage of the ray tracing programmable framework OptiX, through the PlotOptiX API, optimized for massively parallel processing on GPUs. As a result, fast results can be obtained. Three different cases were analyzed to benchmark the code with a commercial tool. A remarkable reduction in time was found when using the proposed code.

One current limitation of the code is that communication between the Python code on the host and the code running on GPU devices occurs after each batch of segments. These data transfers can be reduced when the final algorithms are implemented for production. Nevertheless, the ray tracing core procedures are heavily optimized and are not limiting the efficiency of the overall workflow.

The final step in the workflow, the field calculation, is implemented entirely in Python for clarity of the math formulas and also for ease of experimentation. The code efficiency of this step may be significantly improved with parallelization and optimization in a lower-level language, including possible GPU implementation. Future work also includes the improvement of the algorithms using artificial intelligence and the integration of the code into a powerful tool that is being currently developed by the authors [23].

## Figures and Tables

**Table 1:** Comparison of the required time to run the different test cases.

	Test Case 1	Test Case 2	Test Case 3
Proposed code	36 s	3 s	2 min 53 s
NewFasant	2 min 38 s using 4 processors	28 s using 4 processors	Not able to run it

## Data Availability

Not applicable.

## Appendix A

The received field at a point is calculated as:

$$E_{total}=E_{dir}+E_{ref}+E_{dif}$$

(1) $E_{dir}$ is the field due to the direct ray. The field at the observation point (*O*) due to the direct ray is obtained as follows:

(A1) $$\vec{E}\left(O\right)={\vec{E}}_{t}\left(\theta ,\phi \right)\frac{\exp\left(-jk_{0}r\right)}{r}$$

$k=\frac{2\pi }{\lambda }$

*r* is the distance between transmitter and receiver.

(A2) $${\vec{E}}_{t}\left(\theta ,\phi \right)=\sqrt{\frac{\eta P_{r}G}{4\pi }}{\vec{E}}_{0}\left(\theta ,\phi \right)$$

η=120π.

$P_{r}$ is the power radiated by the transmitting antenna.

*G* is the gain transmitting antenna.

${\vec{E}}_{t}\left(\theta ,\phi \right)$ is the normalized radiation pattern of the transmitting antenna.

(2) $E_{ref}$ is the field due to the reflected rays:

(A3) $$E_{R}\left(r\right)=E_{0}R\frac{e^{-jkr}}{r}.$$

$E_{0}$ is the incident field in the point of reflection. It is calculated using (A1)

*r* is the total distance

(A4) $$R=\prod_{i}R_{i}.$$

$R_{i}$ is the reflection coefficient in each hit. It depends on the polarization.

For soft polarization:

(A5) $$R_{⊥}\left(\psi \right)=\frac{\mathrm{sen}\psi -\sqrt{\varepsilon_{r}-\cos^{2}\psi }}{\mathrm{sen}\psi +\sqrt{\varepsilon_{r}-\cos^{2}\psi }}.$$

For hard polarization:

(A6) $$R_{∥}\left(\psi \right)=\frac{\varepsilon_{r}\mathrm{sen}\psi -\sqrt{\varepsilon_{r}-\cos^{2}\psi }}{\varepsilon_{r}\mathrm{sen}\psi +\sqrt{\varepsilon_{r}-\cos^{2}\psi }}.$$

$\varepsilon_{r}$ depends on the material of the wall.

ψ is the angle when the ray hits the wall.

(3) $E_{dif}$ is the field due to the diffracted rays:

(A7) $$E_{D}=\frac{E_{0}}{s^{\prime }}D\sqrt{\frac{s^{\prime }}{s\left(s^{\prime }+s\right)}}\cdot e^{-jk\left(s+s^{\prime }\right)}$$

$E_{0}$ is the incident field in the point of diffraction. It is calculated using (A1).

$S^{\prime }$ is the distance traveled by the ray before hitting the wedge.

*S* is the distance traveled by the ray after hitting the wedge.

$D=D_{s}$ if polarization is soft and $D=D_{h}$ if polarization is hard:

(A8) $$\begin{array}{cc} \; & D_{s}=\frac{-\exp(-j\pi /4)}{2n\sqrt{2\pi k}\mathrm{sen}\beta_{0}}\{\\ \; & \cot\left[\frac{\pi +\left(\phi -\phi^{\prime }\right)}{2n}\right]F\left[kLa^{+}\left(\phi -\phi^{\prime }\right)\right]+\\ \; & \cot\left[\frac{\pi -\left(\phi -\phi^{\prime }\right)}{2n}\right]F\left[kLa^{-}\left(\phi -\phi^{\prime }\right)\right]+\\ \; & R_{0}^{⊥}\cot\left[\frac{\pi -\left(\phi +\phi^{\prime }\right)}{2n}\right]F\left[kLa^{-}\left(\phi +\phi^{\prime }\right)\right]+\\ \; & \left.R_{n}^{⊥}\cot\left[\frac{\pi +\left(\phi +\phi^{\prime }\right)}{2n}\right]F\left[kLa^{+}\left(\phi +\phi^{\prime }\right)\right]\right\}. \end{array}$$

(A9) $$\begin{array}{cc} \; & D_{h}=\frac{-\exp(-j\pi /4)}{2n\sqrt{2\pi k}\mathrm{sen}\beta_{0}}\{\\ \; & \cot\left[\frac{\pi +\left(\phi -\phi^{\prime }\right)}{2n}\right]F\left[kLa^{+}\left(\phi -\phi^{\prime }\right)\right]+\\ \; & \cot\left[\frac{\pi -\left(\phi -\phi^{\prime }\right)}{2n}\right]F\left[kLa^{-}\left(\phi -\phi^{\prime }\right)\right]+\\ \; & R_{0}^{∥}\cot\left[\frac{\pi -\left(\phi +\phi^{\prime }\right)}{2n}\right]F\left[kLa^{-}\left(\phi +\phi^{\prime }\right)\right]+\\ \; & \left.R_{n}^{∥}\cot\left[\frac{\pi +\left(\phi +\phi^{\prime }\right)}{2n}\right]F\left[kLa^{+}\left(\phi +\phi^{\prime }\right)\right]\right\}. \end{array}$$

n=2−α/π, where α is the angle of the wedge.

Vertical angle: $\beta_{0}^{\prime }=\beta_{0}$.

Horizontal incoming angle: $\phi^{\prime }$.

Horizontal outcoming angle: ϕ.

(A10) $$L=\frac{ss^{\prime }}{s+s^{\prime }}{\mathrm{sen}}^{2}\beta_{0}.$$

(A11) $$a^{\pm }\left(\chi \right)=2\cos^{2}\left(\frac{2n\pi N^{\pm }-\chi }{2}\right),\chi =\phi \pm \phi^{\prime }.$$

(A12) $$F\left(x\right)=j\sqrt{2\pi }\sqrt{x}\exp\left(jx\right)\left\{\left[\frac{1}{2}-C\left(\sqrt{\frac{2}{\pi }}\sqrt{x}\right)\right]-j\left[\frac{1}{2}-S\left(\sqrt{\frac{2}{\pi }}\sqrt{x}\right)\right]\right\}.$$

(A13) $$\begin{array}{cc} \; & S\left(x\right)=\int_{0}^{x}\mathrm{sen}\left(\frac{\pi }{2}\tau^{2}\right)d\tau \\ \; & C\left(x\right)=\int_{0}^{x}\cos\left(\frac{\pi }{2}\tau^{2}\right)d\tau . \end{array}$$

$R_{0}^{⊥}$ is calculated using

(A14) $$R_{⊥}\left(\psi \right)=\frac{\mathrm{sen}\psi -\sqrt{\varepsilon_{r}-\cos^{2}\psi }}{\mathrm{sen}\psi +\sqrt{\varepsilon_{r}-\cos^{2}\psi }}$$

and considering that $\psi =\phi^{\prime }$.

$R_{n}^{⊥}$ is calculated using

(A15) $$R_{⊥}\left(\psi \right)=\frac{\mathrm{sen}\psi -\sqrt{\varepsilon_{r}-\cos^{2}\psi }}{\mathrm{sen}\psi +\sqrt{\varepsilon_{r}-\cos^{2}\psi }}$$

and considering that ψ=nπ−ϕ.

$R_{0}^{∥}$ is calculated using

(A16) $$R_{∥}\left(\psi \right)=\frac{\varepsilon_{r}\mathrm{sen}\psi -\sqrt{\varepsilon_{r}-\cos^{2}\psi }}{\varepsilon_{r}\mathrm{sen}\psi +\sqrt{\varepsilon_{r}-\cos^{2}\psi }}$$

and considering that $\psi =\phi^{\prime }$.

$R_{n}^{∥}$ is calculated using

(A17) $$R_{∥}\left(\psi \right)=\frac{\varepsilon_{r}\mathrm{sen}\psi -\sqrt{\varepsilon_{r}-\cos^{2}\psi }}{\varepsilon_{r}\mathrm{sen}\psi +\sqrt{\varepsilon_{r}-\cos^{2}\psi }}$$

and considering that ψ=nπ−ϕ.
